# Regulation of Fibre Contraction in a Rat Model of Myocardial Ischemia

**DOI:** 10.1371/journal.pone.0009528

**Published:** 2010-03-04

**Authors:** Young Soo Han, Ozgur Ogut

**Affiliations:** Division of Cardiovascular Diseases, Mayo Clinic, Rochester, Minnesota, United States of America; University of Cincinnati, United States of America

## Abstract

**Background:**

The changes in the actomyosin crossbridge cycle underlying altered contractility of the heart are not well described, despite their importance to devising rational treatment approaches.

**Methodology/Principal Findings:**

A rat ischemia–reperfusion model was used to determine the transitions of the crossbridge cycle impacted during ischemia. Compared to perfused hearts, the maximum force per cross-sectional area and Ca^2+^ sensitivity of fibers from ischemic hearts were both reduced. Muscle activation by photolytic release of Ca^2+^ and ATP suggested that the altered contractility was best described as a reduction in the rate of activation of noncycling actomyosin crossbridges to activated, cycling states. More specifically, the apparent forward rate constant of the transition between the nonforce bearing A-M.ADP.Pi state and the bound, force bearing AM*.ADP.Pi state was reduced in ischemic fibers, suggesting that this transition is commensurate with initial crossbridge activation. These results suggested an alteration in the relationship between the activation of thin filament regulatory units and initial crossbridge attachment, prompting an examination of the post-translational state of troponin (Tn) T and I. These analyses indicated a reduction in the diphosphorylated form of TnT during ischemia, along with lower Ser23/24 phosphorylation of TnI. Treatment of perfused fibers by 8-Br-cAMP increased Ser23/24 phosphorylation of TnI, altering the reverse rate constant of the Pi isomerization in a manner consistent with the lusitropic effect of β-adrenergic stimulation. However, similar treatment of ischemic fibers did not change TnI phosphorylation or the kinetics of the Pi isomerization.

**Conclusions:**

Ischemia reduces the isomerization from A-M.ADP.Pi to AM*.ADP.Pi, altering the kinetics of crossbridge activation through a mechanism that may be mediated by altered TnT and TnI phosphorylation.

## Introduction

Cardiac muscle activation is regulated by the troponin complex, which confers Ca^2+^ sensitivity to the actomyosin (AM) crossbridge cycle responsible for force production [1]. Under normal physiological conditions, crossbridge cycling models suggest that cardiac muscle actomyosin may produce force in at least two distinct steps: i) a transition between two post-ATP hydrolysis states, A-M.ADP.Pi 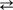 AM*.ADP.Pi and ii) release of Pi from the AM*.ADP.Pi state to yield AM*.ADP [2]–[7]. With a transient or chronic reduction of blood flow to the heart, cardiac muscle force production is impaired regardless of ATP or Ca^2+^ availability, suggesting that the depression of force is produced in part by intrinsic changes to the contractile filaments [8]–[14]. These alterations to the contractile filaments involve both thick and thin filament proteins, and additionally implicate accompanying alterations in the upstream signaling mechanisms [15]–[17]. Therefore, the relationship between altered contractile protein phosphorylation and the actomyosin crossbridge cycle is of significant interest in defining the mechanisms leading to reduced contractility with altered blood flow.

To determine the transitions of the actomyosin crossbridge cycle altered by reduced blood flow, we examined the impact of the ATP hydrolysis products ADP and Pi on steady state and transient force production of fibers prepared from an *in vivo* ischemia – reperfusion model [11]. The effect of Pi on ischemic fibers was quantitatively unique compared to perfused and ischemia – reperfused fibers, suggesting that Pi-bound states of the actomyosin crossbridge cycle were differentially impacted with ischemia. Transient kinetics examined by flash photolysis suggest that the initial rate of crossbridge attachment is reduced, resulting in a smaller number of active heads during ischemia. The reduction in contractility during ischemia was accompanied by changes in the phosphorylation states of the troponin subunits troponin T and I (TnT, TnI). These results provide further insight into the mechanisms behind reduced contractility during ischemia, highlighting the close relationship between thin filament protein phosphorylation and the actomyosin crossbridge cycle.

## Materials and Methods

### Rat Model of Ischemia – Reperfusion and Fiber Preparation

This model was described in detail previously [11], following a protocol approved by the Institutional Animal Care and Use Committee of the Mayo Clinic. Briefly, adult male Sprague-Dawley rats (body wt. ∼250−350 g) were anesthetized using a mixture of ketamine and xylazine (5∶2; 0.5−0.7 mL/kg) administered intramuscularly. The heart was exposed by a midline thoracotomy, and a ligature was placed near the bifurcation of the left coronary artery, restricting flow through the left anterior descending and circumflex artery. The ends were exteriorized and passed through polyethylene tubing. Coronary occlusion was achieved by pressing the tube against the heart muscle while pulling the ligature. Reflow was initiated by releasing the ligature. The following experimental conditions were tested: 90 minutes of perfusion, 30 minutes of occlusion (ischemia), or 30 minutes of ischemia followed by 60 minutes of reperfusion. Following the procedure, the anterolateral papillary muscle was recovered, as its perfusion is dependent on blood flow through the branches of the left coronary [18]. The anterolateral papillary muscle was gently teased into thin strips in pCa8.0 (1×10^−8^ M free Ca^2+^) relaxing solution while on ice. The fibers were typically <250 µm in diameter and <1.5 mm in length. Prepared fibers were permeabilized for 2 h at 4°C with pCa8.0 relaxing solution containing 1% Triton X-100. To test the impact of cAMP-dependent protein kinase (PKA) activation on perfused and ischemic anterolateral papillary muscle fibers, the cell permeable analog 8-Br-cAMP (1 mM, Calbiochem) was included during the permeabilization step.

### Experimental Setup and Protocols

The workstation to test permeabilized fiber contraction has been described previously [11], and employs an AE801 force transducer (SensoNor, Norway) opposite a model 312C length controller (0.25 ms length step response time; Aurora Scientific, Canada). The ends of the permeabilized fibers are fixed with glutaraldehyde, followed by attachment of aluminum T-clips [11]. Measurements based on controlling the length of the fiber were programmed using the 600A Controller software (Aurora Scientific) and data were typically collected at 1000 Hz. Temperature of the solutions during experiments was maintained at 15±0.1°C. Solutions to activate and relax fibers contained 5 mM free MgATP, 1 mM free Mg^2+^, 10 mM total EGTA, 25 mM total BES, pH 7, and when appropriate, 25 mM creatine phosphate and 15 units/mL creatine kinase. Ionic strength was maintained at 200 mM using potassium methanesulphonate. Activating solutions of varying free Ca^2+^ were prepared using an iterative program [11]. For experiments testing the effect of ADP, 1 mM total ADP was included in the activating solutions while maintaining ionic strength at 200 mM. For experiments testing the effect of phosphate, varying volumes of 1 M phosphate buffer, pH 7, were added to ensure the desired final concentration, with appropriate adjustments to maintain ionic strength at 200 mM. All chemicals were the highest grade from Sigma, except for ATP (Special Quality, <0.5% ADP, Roche) and photolysis compounds (Invitrogen).

To test fiber mechanics, an initial control contraction was recorded from each fiber by changing the bathing solution from pCa8.0 to pCa4.0. To measure the force-Ca^2+^ relationship, the fibers were activated with solutions of varying free [Ca^2+^] between pCa8.0 and 4.0. For the measurement of the influence of ADP or Pi on the force-Ca^2+^ relationship, a separate batch of free [Ca^2+^] solutions were mixed including either 1 mM added ADP or 5 mM added Pi. All forces were normalized to cross-sectional area with the assumption that fibers were elliptical in cross-section. Data were fit using the Hill equation to determine the concentration of free Ca^2+^ required for half-maximal force (EC50). Fiber stiffness was measured using four consecutive fiber length releases of 0.05, 0.1, 0.15 and 0.2% [19]. The change in force per cross-sectional area per mm change in length was plotted, and the slope of the linear fit was recorded as the stiffness. To measure the sensitivity of force redevelopment to increasing Pi, fibers were activated by pCa 4.5, followed by pCa4.5 solution containing 5, 10, 20 and 30 mM total Pi. Ionic strength was maintained at 200 mM for all Pi concentrations, although for 30 mM Pi this necessitated decreasing the total creatine phosphate concentration from 25 mM to 17.5 mM. The rate constant of force redevelopment after a length release (k_F_) was measured by a single exponential fit of the force recovery profile following a 5% decrease in fiber length within 3 ms [7]. All data are presented as average ± SEM. Statistical comparisons between groups were performed by Student's *t*-test using a Bonferroni adjustment when multiple comparisons were necessary between three surgery groups.

### Muscle Activation by Flash Photolysis

The rate constant of force development upon flash photolysis of NP-EGTA (k_Act_) was determined for perfused, ischemic and ischemia-reperfused fibers. The fiber was placed in a pCa8.0 relaxing solution that was prepared as before except for the substitution of 20 mM PIPES for BES and inclusion of 10 mM dithiothreitol [20]. This was followed by incubation in a pre-activating solution that reduced EGTA to 0.1 mM. Subsequently, the fiber was allowed to equilibrate in a pCa6.2 solution containing NP-EGTA (2 mM) and then briefly suspended in air for flash photolysis using a Xe flash lamp system (Model JML-C2; Rapp OptoElectronic, Germany). Following data recording (<1 s) the fibers were immediately returned to relaxing solution. The ATP-dependent rate constant of force development from Ca^2+^-rigor (k_ATP_) was measured by flash photolysis of NPE-ATP. A fiber in pCa8.0 relaxing solution was transferred to pCa8.0 rigor solution (0 mM ATP) including 1 unit/mL apyrase [21], and then transferred to pCa4.5 rigor solution. Following apyrase incubation, the fiber was placed in pCa4.5 rigor solution containing 5 mM NPE-ATP and 10 mM dithiothreitol. There was no change in force when fibers were placed into the NPE-ATP containing rigor solution. The fiber was then suspended in air for flash photolysis and data recording. The rate constants, k_Act_ and k_ATP_, were determined from the force transients by single exponential fits.

### Troponin T and Myosin Light Chain Phosphorylation Analysis

Two-dimensional SDS-PAGE was used to resolve TnT and myosin light chain (MLC)-1 and -2 phosphoforms essentially as described [22]. Proteins were extracted from the anterolateral papillary muscle by homogenization on ice in a micro tissue grinder using a buffer of 9 M urea, 4% (w/v) 3-([3-cholamidopropyl] dimethylammonio)-2-hydroxy-1-propanesulfonate (CHAPS), 0.5% (v/v) pH 3–10 immobilized pH gradient (IPG) buffer, 1 mM EDTA, and EDTA-free Complete Protease Inhibitor (Roche, Indianapolis, IN). Following homogenization, the insoluble debris was cleared by centrifugation and the supernatant was processed with the 2D Clean-Up Kit (GE Lifesciences). Homogenates were then added to a rehydration solution containing 9 M urea, 2% (w/v) CHAPS, 0.5% (v/v) 3.5 - 5 IPG buffer, 0.002% (w/v) bromophenol blue and protease inhibitor and used to rehydrate 7 cm pH 3-5.6NL IPG gel strips. The IPG strips were focused in the “face-up” mode on an Ettan IPGphor II Isoelectric Focusing Unit. After the first-dimension, the gel strips were consecutively equilibrated for 15 minutes in 6 M urea, 50 mM Bis-Tris, pH 6.4, 30% glycerol, 2% SDS, and 0.002% bromophenol blue containing first 10 mM dithiothreitol and then 2.5% (w/v) iodoacetamide. Proteins on equilibrated IPG gel strips were then resolved by second dimension SDS-PAGE using the Bis-Tris buffering system (Invitrogen, LaJolla, CA). Resolved gels were stained by Deep Purple total protein fluorescent stain, scanned using a Typhoon 9410 imager and analyzed by ImageQuant TL software. As previously described, >95% of the TnT exists as mono- and diphosphorylated forms of the highly abundant low Mr isoform [22], [23], and therefore the analysis focused on these two phosphoforms.

### Ser23/24 Phosphorylation of TnI

To measure the extent of Ser23/24 phosphorylation of TnI, a multiplex Western blotting strategy was used. Samples resolved by SDS-PAGE were transferred to PVDF membrane and incubated simultaneously with a mouse anti-cardiac muscle TnI monoclonal antibody (10T79E, Fitzgerald, Concord, MA) and a phospho-Ser23/24 TnI rabbit polyclonal antibody (Cell Signaling Technology, Danvers, MA). Following washing, the blots were incubated simultaneously with Cy3 labeled anti-mouse IgG and Cy5 labeled anti-rabbit IgG (GE Lifesciences). Blots were scanned on a Typhoon 9410 imager at the appropriate channels, and the scanned images were analyzed using ImageQuant TL software. The relative extent of Ser23/24 phosphorylation of TnI was determined to be the Ser23/24 phosphospecific antibody signal divided by the total TnI mAb signal for each sample. To determine the extent of Ser23/24 phosphorylation of TnI in perfused versus ischemic hearts, homogenates of the anterolateral papillary muscle from five perfused hearts and five ischemic hearts were resolved on a single gel and blotted. The extent of Ser23/24 phosphorylation in the ischemic samples was reported relative to the perfused samples. To determine the effect of 1 mM 8-Br-cAMP treatment on Ser23/24 phosphorylation of TnI in perfused and ischemic rat hearts, ten fibers from the anterolateral papillary muscle were dispersed into two groups, with one group receiving 8-Br-cAMP treatment during permeabilization and the other group remaining untreated. All ten samples from each individual rat were resolved on a single gel and Western blotted to measure the extent of Ser23/24 phosphorylation of TnI. The extent of phosphorylation in the five treated samples was reported relative to the untreated samples. The experiments were repeated with an independent pair of rats, resulting in measurements from two perfused rat hearts and two ischemic rat hearts.

### Phosphorylation State of Myosin Binding Protein-C

The overall phosphorylation state of the thick filament associated protein myosin binding protein-C (MYBP-C) was examined by sequential Pro-Q Diamond phosphoprotein and Deep Purple total protein staining as previously described [22]. Homogenates from perfused and ischemic anterolateral papillary muscles were resolved by 29∶1 8% SDS-PAGE and initially stained with Pro-Q Diamond phosphoprotein stain to measure an index of phosphorylation. The gels were subsequently stained with Deep Purple total protein stain to allow for normalization of the Pro-Q Diamond signal according to total MYBP-C content.

## Results

### Ischemia-Reperfusion Reduces Force and Ca^2+^ Sensitivity

An *in vivo* rat model was used to determine the effect of myocardial ischemia-reperfusion on the maximum force per cross-sectional area (Fmax), maximum fiber stiffness or the force – Ca^2+^ relationship of permeabilized small fiber bundles isolated from the anterolateral papillary muscle. Following thirty minutes of ischemia, Fmax fell from 50.1±2.5 mN/mm^2^ (n = 12) to 36.1±2.4 mN/mm^2^ (n = 12; Table 1). A subset of the fibers were used to determine the [Ca^2+^] required to achieve half-maximal force (EC50), which increased significantly in ischemic fibers to 3.61±0.11 µM (n = 8) compared to 3.32±0.13 µM in perfused fibers (n = 7). This loss of force during ischemia was partially recovered by reperfusion to 41.3±5.2 mN/mm^2^ (n = 5) whereas the EC50 of reperfused fibers was 3.57±0.12 µM (n = 5). The Hill coefficients were not different for any of the conditions tested (data not shown). To determine if the change in Fmax correlated with a change in crossbridge attachment, we measured maximum stiffness per cross-sectional area based on the assumption that measured changes in stiffness would be proportional to the number of attached crossbridges [24]. Similar to the changes in Fmax, stiffness was significantly reduced from 465.8±63.5 mN/mm^2^/mm (n = 7) to 350.2±40.5 mN/mm^2^/mm (n = 8) with ischemia (*P*<0.016), and there was not full recovery with reperfusion (408.2±62.1 mN/mm^2^/mm, n = 5).

**Table 1:** The concentrations of free Ca^2+^ required for half-maximal force (EC_50_) and the maximal Ca^2+^-activated force per cross-sectional area (Fmax) for three surgery groups under varying experimental conditions.

		Perfused	Ischemic	Reperfused
**EC50** (µM)	Control	3.32±0.13 (n = 7)	3.61±0.11* (n = 8)	3.57±0.12 (n = 5)
	5 mM Pi	4.52±0.12 (n = 6)	3.89±0.16† (n = 14)	4.87±0.14 (n = 10)
	1 mM ADP	2.27±0.18 (n = 10)	2.00±0.20 (n = 13)	2.25±0.18 (n = 7)
**Fmax** (mN/mm^2^)	Control	50.1±2.5 (n = 12)	36.1±2.4* (n = 12)	41.3±5.2 (n = 5)
	5 mM Pi	35.9±3.0 (n = 6)	38.3±2.7 (n = 14)	26.0±1.6‡ (n = 10)
	1 mM ADP	42.2±3.1 (n = 10)	35.2±2.6 (n = 13)	35.5±2.9 (n = 7)

Data are presented as average ± S.E.M.

*: denotes *P*<0.016 compared to perfused fibers under identical experimental conditions.

†: denotes *P*<0.016 compared to perfused and reperfused fibers under identical experimental conditions.

‡: denotes *P*<0.016 compared to perfused and ischemic fibers under identical experimental conditions.

### Effect of ADP and Pi on the Force-Ca^2+^ Relationship of Ischemia-Reperfused Fibers

We probed the ADP-dependent transition of the actomyosin crossbridge cycle by steady state measurements of the force - Ca^2+^ relationship of perfused, ischemic and ischemia - reperfused fibers in the presence of 1 mM added ADP. In agreement with previous studies, the addition of 1 mM ADP decreased the EC50 of fibers from the three surgery groups [25], [26]. However, the EC50 values of perfused, ischemic and reperfused fibers in the presence of 1 mM total ADP were not significantly different from each other. The impact of 1 mM total ADP on Fmax was not significant for any of the surgery conditions, in general agreement with prior reports suggesting very modest changes to Fmax at these ADP concentrations [25], [27].

**Figure 1:**
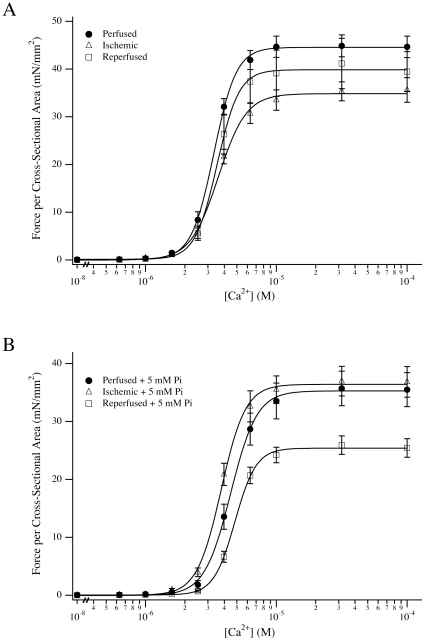
Force-Ca^2+^ relationships of ischemia – reperfused fibers. (A) Force per cross-sectional area versus Ca^2+^ concentration for perfused (•), ischemic (▵) and ischemia-reperfused (□) fibers versus control conditions. (B) Absolute force per cross-sectional area versus Ca^2+^concentration for perfused, ischemic and ischemia – reperfused fibers in the presence of 5 mM added Pi. Data plotted are average ± S.E.M., and the averaged data are fit by the Hill equation for illustrative purposes. Accumulated data from all fibers are presented in Table 1.

The phosphate-bound transitions of the crossbridge cycle were examined at steady state by similar experiments. As expected, the addition of 5 mM Pi decreased the Fmax of perfused fibers from 50.1±2.5 mN/mm^2^ to 35.9±3.0 mN/mm^2^ [4], [5], [25]. The predictable force response of perfused fibers to exogenous Pi was accompanied by a general rightward shift of the EC50 of force activation (Fig. 1B). Surprisingly, ischemic fibers demonstrated a significantly different response to 5 mM Pi. The EC50 of ischemic fibers in the presence of Pi (3.89±0.16 µM) was significantly different when compared to perfused (4.52±0.12 µM) and ischemia-reperfused (4.87±0.14 µM) fibers. The depressive effect of 5 mM Pi on force was recovered with reperfusion, reducing Fmax by 37% to 26.0±1.6 mN/mm^2^.

### Effect of Increasing Pi on F_max_ and the Rate Constant of Force Redevelopment

**Figure 2:**
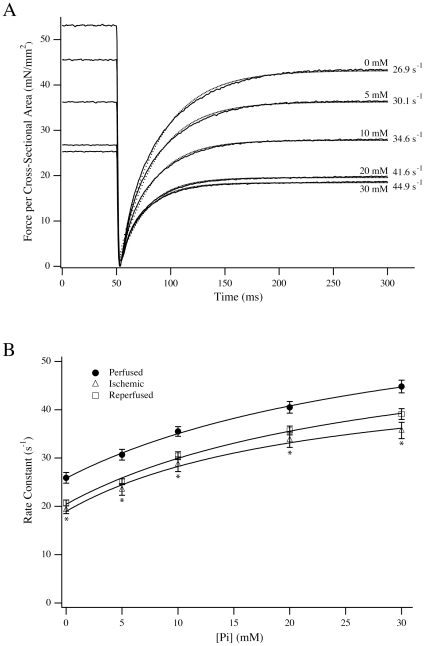
Force redevelopment following length release. (A) Representative traces of force after length release for a perfused fiber activated by pCa4.5 solution including varying concentrations of added Pi. The force recovery transients were analyzed by single exponential fits (dotted lines) to determine the rate constant of force redevelopment. (B) The rate constant of force redevelopment for perfused (•; n = 7), ischemic (▵; n = 10) and ischemia-reperfused (□; n = 7) fibers. Data presented are average ± S.E.M. * *P*<0.016 compared to perfused.

We measured the effect of Pi on the Fmax of perfused, ischemic and ischemia-reperfused fibers to further explore the steady state response to exogenous Pi. We hypothesized that if the relative insensitivity of ischemic fibers to 5 mM Pi is reflective of a change in the rate of the transitions between Pi-bound states, then the effect should manifest at other Pi concentrations. Fibers from a new cohort of rats were activated at pCa4.5 and then moved to pCa4.5 solutions containing 20 and 30 mM Pi. When compared to zero added Pi, relative force declined to 61.3±1.7% (n = 14) of maximum in ischemic fibers as compared to 54.2±0.9% (n = 17) and 50.0±1.9% (n = 12) in perfused and reperfused fibers. This relationship was similar at 30 mM Pi, wherein the relative force maintained was 51.3±1.7% (n = 14) in ischemic fibers as compared to 41.9±0.9% (n = 17) and 40.0±1.5% (n = 12) in perfused and reperfused fibers, respectively. Under all cases, the maintained relative force in ischemic fibers was significantly higher than for perfused and reperfused fibers (*P*<0.016). These data were complemented by measurements of the rate constant of force redevelopment, k_F_, following a length release [7]. At zero added Pi, k_F_ was significantly reduced in ischemic (19.3±0.8 s^−1^) and ischemia – reperfused fibers (20.7±0.6 s^−1^) as compared to perfused fibers (25.9±1.1 s^−1^). For all fibers, increasing [Pi] increased the rate of force redevelopment, but ischemic fibers had significantly reduced rates at all Pi concentrations tested, whereas ischemia – reperfused fibers were intermediate (Fig. 2). We fit the Pi dependence of the force redevelopment using the relationship k_F_ = k_1_+(k_−1_ [Pi])/(K2+[Pi]) according to Scheme 1 [3], [7]. In this scheme, the initial attachment is commensurate with the Pi isomerization step (k_1_/k_−1_) whereas K2 is the equilibrium constant for the Pi release step:
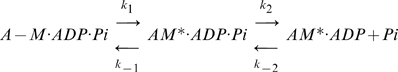
Scheme 1

In this scheme, the necessary transition that activates crossbridges from non-cycling to cycling states [28] is implicit in the first step. In ischemic fibers, both k_1_ and k_−1_ were reduced (18.9±0.8 s^−1^ and 30.7±1.7 s^−1^ respectively) versus perfused fibers (26.6±0.8 s^−1^ and 42.3±3.1 s^−1^; *P*<0.016 for both comparisons). Values were intermediate for ischemia – reperfused fibers where k_1_ was 20.4±0.6 s^−1^ and k_−1_ was 37.4±2.9 s^−1^. The value of K2 determined from the fits was 39.4±4.6 mM for perfused fibers, which was significantly higher than in ischemic fibers (24.5±3.2 mM; *P*<0.016) but not different from ischemia-reperfused fibers (29.4±3.3 mM).

### Activation by Flash Photolysis

**Figure 3:**
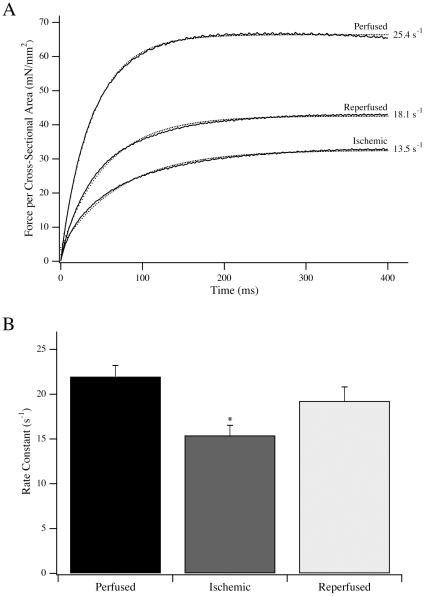
Activation of fiber contraction by Ca^2+^. (A) Typical force transients from representative perfused, ischemic and ischemia-reperfused fibers activated by Ca^2+^ release following flash photolysis of NP-EGTA. Single exponential fits to the data traces are shown as dotted lines, along with the rate constant of force activation. (B) The rate constant of force activation for perfused (n = 11), ischemic (n = 10) and ischemia – reperfused (n = 9) fibers activated by flash photolysis of NP-EGTA. Data are presented as average ± S.E.M. * *P*<0.016 compared to perfused fibers.

**Figure 4:**
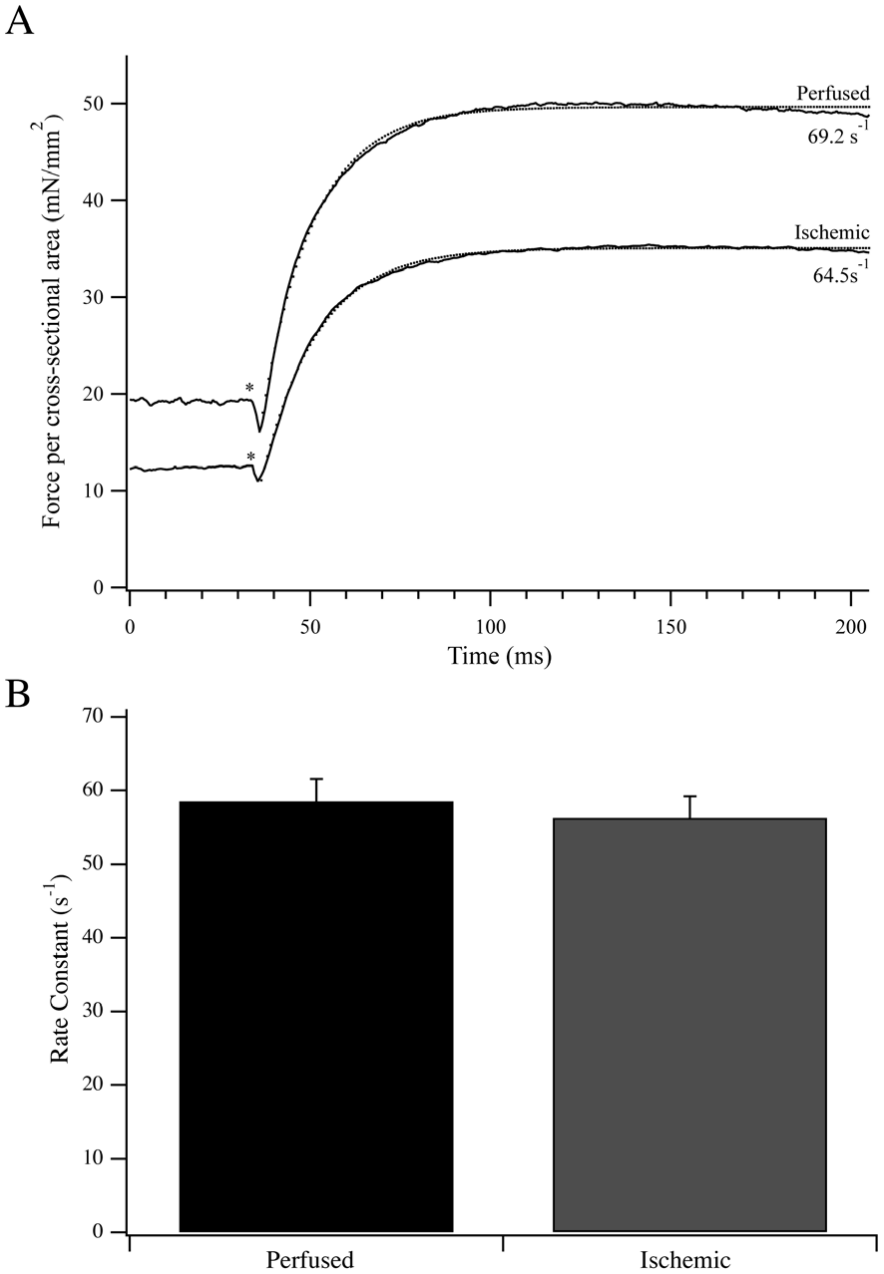
Activation of fiber contraction by flash photolysis of NPE-ATP. (A) Typical force transients from a representative perfused and ischemic fiber activated from Ca^2+^-rigor by photoliberation of ATP from NPE-ATP. Single exponential fits to the data traces are shown as dotted lines along with the rate constant of force activation. (B) The rate constant of force activation for perfused (n = 13) and ischemic (n = 12) fibers activated by flash photolysis of NPE-ATP. Data presented are average ± S.E.M.

To complement the steady state measurements, we measured the activation kinetics of fibers in response to flash photolysis of NP-EGTA (Fig. 3) and NPE-ATP (Fig. 4) in a new cohort of rats. Activation of perfused fibers by free Ca^2+^ release following flash photolysis of NP-EGTA resulted in a rapid rise in force, with a maximum value of 58.0±4.2 mN/mm^2^ and a rate constant of 22.0±1.2 s^−1^ (n = 11). Both the force produced and k_Act_ were significantly reduced in ischemic fibers to 33.6±2.9 mN/mm^2^ and 15.4±1.1 s^−1^, respectively (n = 10; *P*<0.016). For reperfused fibers, the force produced and rate constant of activation were intermediate at 42.1±1.6 mN/mm^2^ and 19.3±1.6 s^−1^ (n = 9). As the major difference in rate and force was observed between perfused and ischemic fibers, the NP-EGTA activation data were complemented with NPE-ATP activation data for perfused and ischemic fibers in Ca^2+^-rigor. Force produced by transferring perfused and ischemic fibers from relaxing to Ca^2+^-rigor solution was 18.4±2.2 mN/mm^2^ and 11.9±1.5 mN/mm^2^, respectively (*P*<0.05). Following photolysis of NPE-ATP, there was a small, brief drop in force prior to force activation (Fig. 4; [29], [30]). The additional active force produced from the Ca^2+^-rigor level was 37.2±3.8 mN/mm^2^ and 23.4±2.2 mN/mm^2^ for perfused and ischemic fibers, respectively (*P*<0.05). Total force produced (Ca^2+^-rigor + active) in these experiments was 55.6±5.8 mN/mm^2^ (n = 13) for perfused fibers and 35.3±3.5 mN/mm^2^ (n = 12) for ischemic fibers. However the rate constant of force activation from Ca^2+^-rigor, k_ATP_, was not different between perfused and ischemic fibers, measuring 58.5±3.0 s^−1^ (n = 13) and 56.3±2.9 s^−1^ (n = 12) respectively (Fig. 4). Notably, the rate constants of force activation from the Ca^2+^-rigor state by caged ATP were significantly higher than those resulting from activation due to release of caged-Ca^2+^.

### Myosin Light Chain and Troponin T Phosphorylation during Ischemia

**Figure 5:**
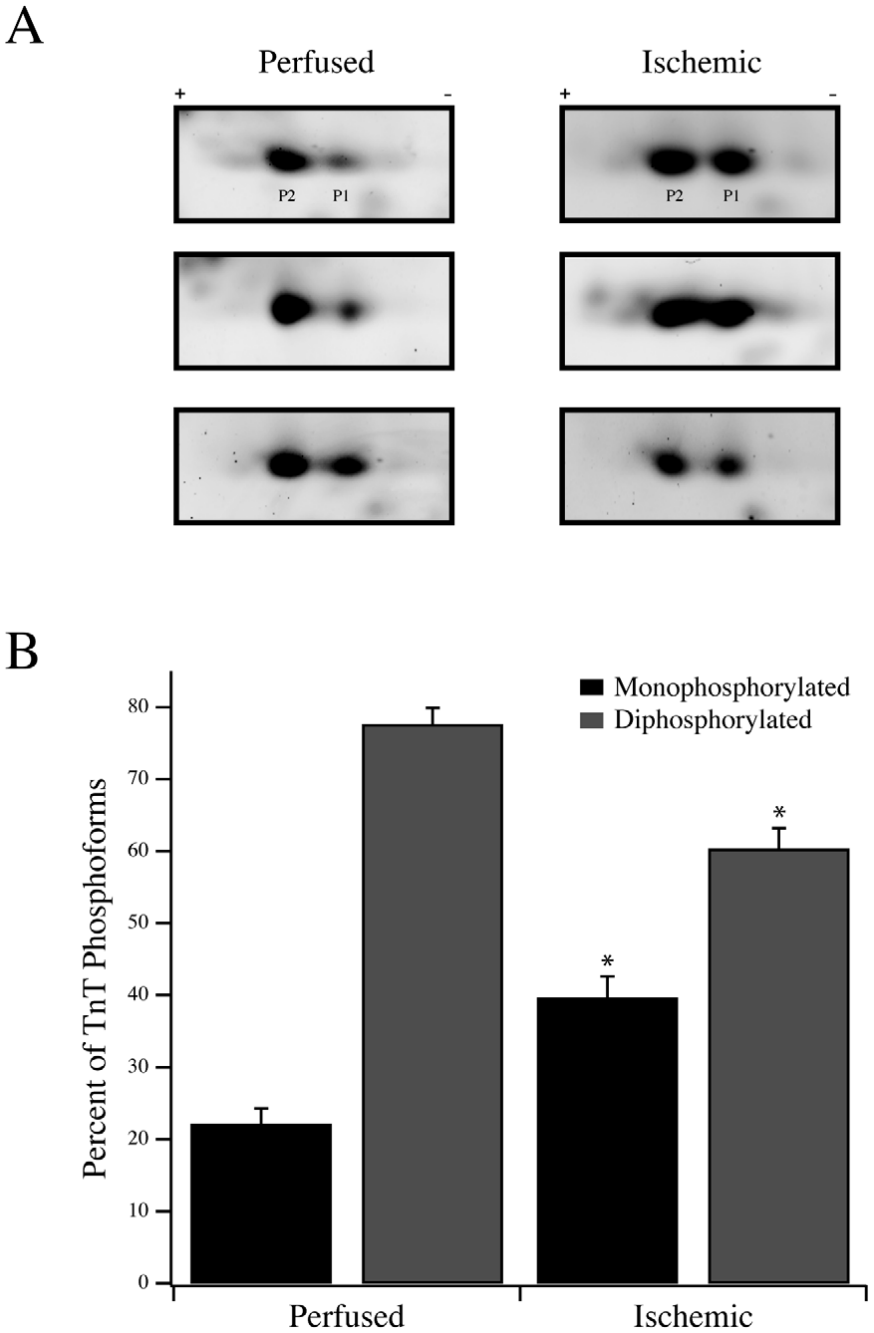
Troponin T phosphorylation in perfused and ischemic fibers. (A) Two-dimensional SDS-PAGE with 3–5.6 NL IPG strips was used to resolve TnT into its phosphoforms. Three representative perfused and ischemic samples are shown with the two predominant phosphoforms labeled P1 (monophosphorylated) and P2 (diphosphorylated). (B) Following densitometric analysis, the percentages of monophosphorylated and diphosphorylated TnT, as a percentage of total TnT phosphoform expression (P1 + P2), are plotted for perfused and ischemic fibers. * *P*<0.05 compared to the corresponding perfused phosphoform.

The levels of phosphorylation of MLC-1 and MLC-2 were determined in perfused and ischemic fibers. Both MLC-1 and MLC-2 were present largely as nonphosphorylated and singly phosphorylated forms [22], [31]. For MLC-1, the nonphosphorylated form was 86.6±2.5% and 86.5±2.9% of total expression in perfused and ischemic fibers, respectively (Table 2). Similarly, MLC-2 was present predominantly in the nonphosphorylated form in perfused (76.3±2.8%) and ischemic (73.1±2.7%) fibers. When comparing the perfused to the ischemic states, there was no statistically significant difference in the level of phosphorylation of either light chain. To assess the phosphorylation state of TnT, two-dimensional SDS-PAGE was used to resolve phosphoforms. As previously demonstrated, rat cardiac TnT is expressed as two isoforms, with the lower Mr isoform being highly abundant [22], [23]. When resolved by two-dimensional SDS-PAGE (Fig. 5A), this abundant lower Mr TnT is expressed predominantly as two phosphoforms that are mono- and diphosphorylated [22]. In perfused rat hearts, the monophosphorylated TnT represented 22.1±2.2% (n = 11) of the phosphoforms whereas the diphosphorylated TnT was 77.6±2.3%. In ischemic fibers, the percentage of monophosphorylated TnT increased significantly to 39.6±3.0% (n = 9; *P*<0.05) and the percentage of diphosphorylated TnT decreased to 60.3±2.9% (Fig. 5B).

**Table 2:** Phosphorylation status of MLC-1 and MLC-2 in perfused and ischemic fibers.

		MLC-1	MLC-2
**Perfused** (n = 8)	0-P	86.6±2.5%	76.3±2.8%
	1-P	13.4±2.5%	23.7±2.8%
**Ischemic** (n = 7)	0-P	86.5±2.9%	73.1±2.7%
	1-P	13.5±2.9%	26.9±2.7%

Data presented are average ± S.E.M.

### TnI and MYBP-C Phosphorylation during Ischemia

**Figure 6:**
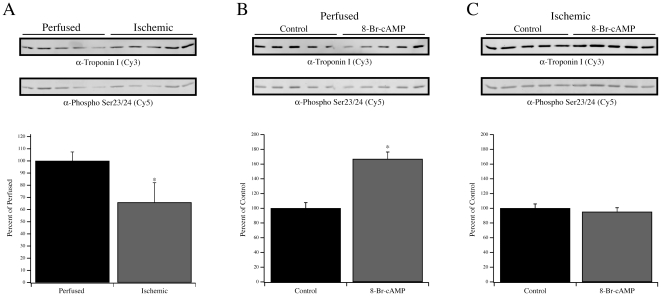
Phosphorylation of TnI at Ser23/24. (A) Multiplex Western blotting to determine the extent of Ser23/24 phosphorylation of TnI in five perfused and five ischemic samples. Resolved samples were simultaneously labeled using a mouse α-TnI monoclonal antibody (Top row; Cy3 labeled secondary antibody detection) and rabbit phospho-Ser23/24 TnI polyclonal antibody (Bottom row; Cy5 labeled secondary antibody detection). Graph represents densitometry analysis of the Ser23/24 phosphorylated TnI signal divided by the total TnI signal. Fibers from a perfused (B) or ischemic (C) rat heart were left untreated or treated with 8-Br-cAMP, followed by multiplex Western blotting for total and Ser23/24 phosphorylated TnI. Data presented are average ± S.E.M. * *P*<0.05 compared to the respective perfused or control condition.

**Figure 7:**
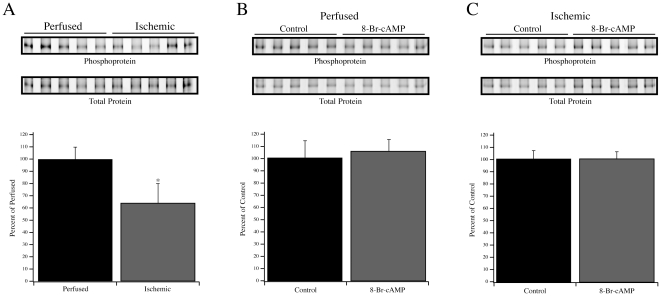
Phosphorylation of MYBP-C. (A) Pro-Q Diamond phosphoprotein staining (Top row) and Deep Purple total protein staining (Bottom row) showing MYBP-C in perfused and ischemic homogenates. The graph represents the phosphoprotein signal divided by the total protein signal. (B) Analyses of MYBP-C phosphorylation in perfused and ischemic (C) fibers with and without 8-Br-cAMP treatment. Data presented are average ± S.E.M. * *P*<0.05 compared to the perfused fibers.

The extent of Ser23/24 phosphorylation of TnI was determined by multiplex Western blotting of perfused and ischemic anterolateral papillary homogenates (Fig. 6A). Relative to the extent of Ser23/24 phosphorylation in perfused hearts (100±7.4%; n = 5), ischemic hearts demonstrated a significant decline in relative Ser23/24 phosphorylation to 65.9±16.2% (n = 5; *P*<0.05). These data were complemented by an examination of the phosphorylation state of MYBP-C as determined by phosphoprotein and total protein staining (Fig. 6A). Relative to the level of MYBP-C phosphorylation in perfused hearts (100±9.8%, n = 5), ischemic hearts demonstrated a significant reduction (63.6±15.9%, n = 5; *P*<0.05). As both TnI and MYBP-C are downstream targets of β-adrenergic signaling [32], it remained possible that the reduction in phosphorylation reflected a reduction of endogenous PKA activity. Therefore, anterolateral papillary muscle fibers from two perfused and two ischemic rat hearts were left untreated or treated with 8-Br-cAMP to activate endogenous PKA. In perfused rat hearts, the relative Ser23/24 phosphorylation level following 8-Br-cAMP treatment increased significantly from the baseline level (166.9±9.5% vs. 100±7.7%, n = 10 fibers from two rats; *P*<0.05). However, 8-Br-cAMP treatment of anterolateral papillary muscle fibers from ischemic rat hearts did not result in an increase in Ser23/24 phosphorylation of TnI. In untreated ischemic fibers, the relative level of phosphorylation was 100±5.9% (n = 10 fibers from two rats) whereas treatment with 8-Br-cAMP resulted in a relative level of 95.2±5.5% (Fig. 6B,C). The impact of 8-Br-cAMP treatment on MYBP-C phosphorylation in perfused and ischemic fibers was also analyzed (Fig. 7B,C). In contrast to TnI, treatment of perfused fibers did not significantly increase the total phosphorylation of MYBP-C (100±13.6% vs. 105.3±9.3%). This was similar to ischemic fibers wherein the total phosphorylation of MYBP-C was not changed by 8-Br-cAMP treatment (100±6.5% vs. 100±5.6%).

**Figure 8:**
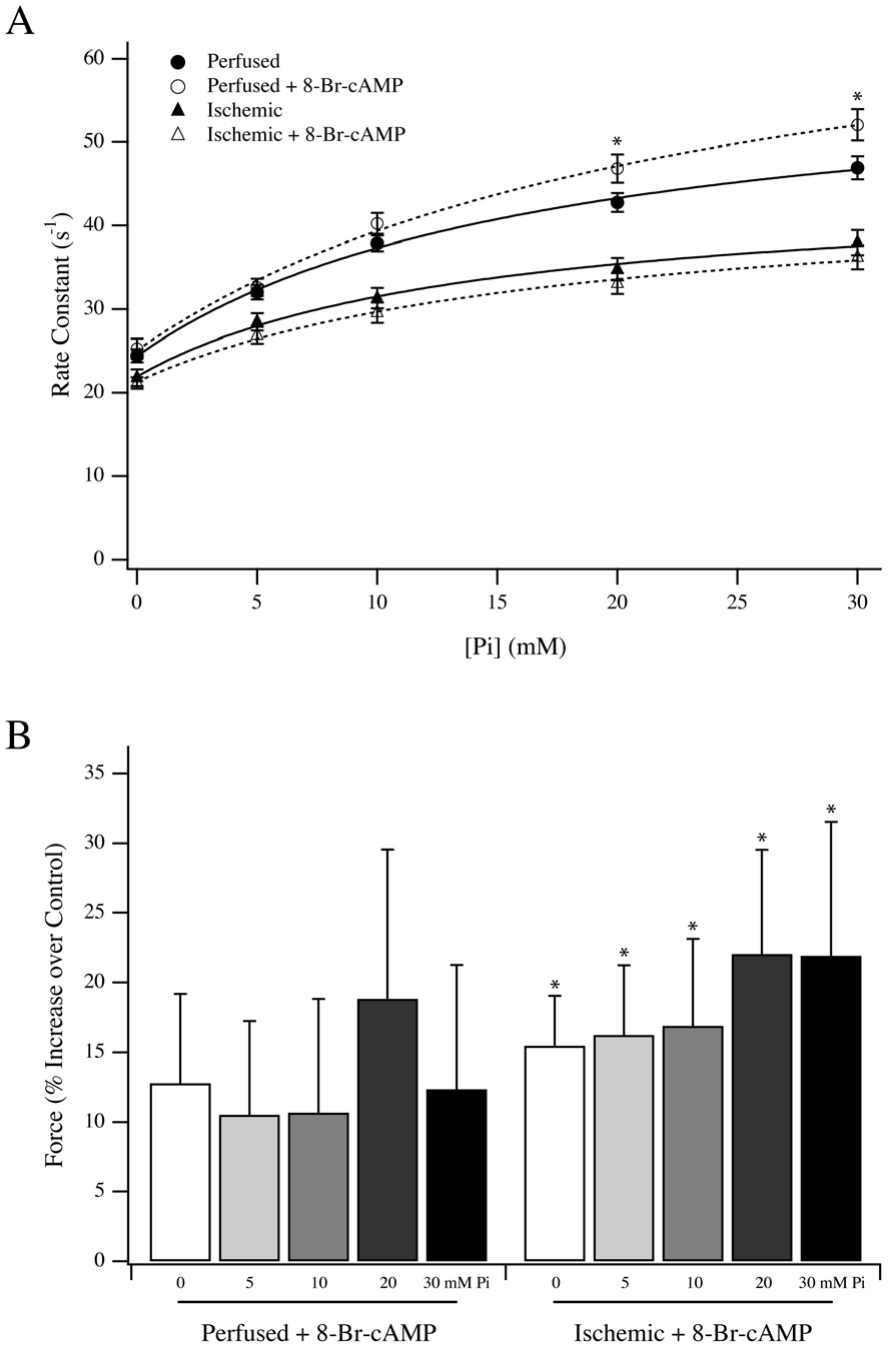
Force and rate constant of force development following 8-Br-cAMP treatment. (A) The rate constant of force redevelopment at various [Pi] for perfused (circles) and ischemic (triangles) fibers, with (open symbols) and without (closed symbols) 8-Br-cAMP treatment. * *P*<0.05 compared to the control perfused condition. (B) The change in the force per cross-sectional area for perfused and ischemic fibers treated with 8-Br-cAMP. Data presented are average ± S.E.M. * *P*<0.05 compared to untreated ischemic fibers.

To determine the impact of 8-Br-cAMP treatment on the Fmax and force redevelopment in the presence of Pi, 8-Br-cAMP treated and untreated fibers from perfused and ischemic rat hearts were examined (Fig. 8). Treatment of perfused fibers with 8-Br-cAMP did not significantly influence k_1_ (24.4±0.8 s^−1^ vs. 25.1±1.1 s^−1^, n = 6 and 7 fibers from 2 rats, respectively) whereas k_−1_ was significantly increased from 36.7±3.0 s^−1^ to 49.7±3.9 s^−1^ (Fig. 7; *P*<0.05). In contrast, neither k_1_ (21.9±1.0 s^−1^ vs 21.3±0.7 s^−1^, n = 9 fibers from 3 rats) nor k_−1_ (23.3±1.5 s^−1^ vs 25.7±2.5 s^−1^) was significantly influenced by 8-Br-cAMP treatment of ischemic fibers. Compared to untreated perfused fibers, 8-Br-cAMP treated perfused fibers trended towards higher Fmax when activated by pCa4.5 in the presence of 0, 5, 10, 20 and 30 mM Pi. In fibers from ischemic rat, the Fmax at all [Pi] increased significantly following 8-Br-cAMP treatment (*P*<0.05).

## Discussion

Deciphering the mechanisms contributing to the decline in contractility of the heart is fundamental to developing new treatment modalities. We have previously demonstrated that permeabilized fibers from ischemic hearts produce submaximal force regardless of maximum ATP or Ca^2+^ availability [11]. However, the underlying causes for the change in contractility, particularly at the level of the actomyosin crossbridge cycle, are not well understood. To further explore this question, we used a rat model to examine the steady state and transient contractile response of permeabilized anterolateral papillary fiber bundles in order to identify the transitions of the actomyosin crossbridge cycle altered with myocardial ischemia. To probe steady state changes in the actomyosin crossbridge cycle, force – [Ca^2+^] relationships were determined in the presence and absence of exogenous Pi and ADP. These products of ATP hydrolysis influenced the force – [Ca^2+^] relationship of fibers generally as expected [25]. The addition of ADP caused a significant Ca^2+^-sensitization of muscle fibers from all surgery groups, although the measured effects were not significantly different between groups. By contrast, Pi decreased the Ca^2+^-sensitivity of force production in all surgery groups. However, ischemic fibers better retained Ca^2+^ sensitivity and force in the presence of 5, 20 and 30 mM Pi when compared to perfused and reperfused fibers. This distinct response suggested that the cellular events associated with ischemia impacted the Pi-bound states of the actomyosin crossbridge cycle. In the presence of Pi, the higher Ca^2+^-sensitivity of ischemic fibers may signal retention of attached crossbridges through near-neighbor thin filament regulatory unit interactions that better preserve the level of activation [25], [28], [33], [34]. The Pi-bound transitions of the crossbridge cycle involve an isomerization between a low/no-force state and an attached force-producing state that precedes Pi release according to Scheme 1 [3]–[7]. The blunted effect of Pi on ischemic fibers is consistent with the preservation of attached crossbridges in the AM*.ADP.Pi and AM*.ADP states, whereas similar [Pi] in perfused fibers drives a larger proportion of attached crossbridges to detached or nonforce producing states [4], [7].

To complement these steady state measurements, we examined transient activation kinetics following photoliberation of Ca^2+^ and ATP. Both the maximum force and the rate constant of force activation with Ca^2+^, k_Act_, were lower for ischemic fibers as compared to perfused fibers (Fig. 3). The rate is unlikely to be limited by the binding of Ca^2+^ to troponin C, as this process is at least an order of magnitude faster than k_Act_ [35], [36]. This leaves k_Act_ to be limited by the rate of initial crossbridge activation and attachment to the thin filament or the intrinsic kinetics of myosin as it cycles between force bearing and non-force bearing states [37]. To better discriminate which of these general processes is impacted with ischemia, we also measured the rate constant of force activation from Ca^2+^-rigor following photoliberation of ATP (Fig. 4). In comparison to the k_Act_ measurement, for which crossbridges begin in detached states, k_ATP_ measures force activation from attached states established during Ca^2+^-rigor. Fibers in Ca^2+^-rigor were expected to bypass the potential rate-limiting effects of initial crossbridge attachment to activated thin filament regulatory units, allowing a measurement of the rate of force activation due directly to the cycling of activated actomyosin crossbridges between force bearing and non-force bearing states. If the physiological processes limiting k_Act_ in ischemic fibers were intrinsic to myosin and its enzymatic ATPase activity, force activation of perfused and ischemic fibers from Ca^2+^-rigor by ATP would be expected to demonstrate different rate constants. However, k_ATP_ was nearly identical for ischemic and perfused fibers, suggesting that the attached myosins in these fibers had similar intrinsic cycling kinetics. We also noted that the ratio of force produced following photoliberation of ATP to Ca^2+^-rigor force was ∼2∶1 in both perfused and ischemic fibers, suggesting that a change in the force generated per crossbridge was unlikely. Even when prepared from failing, explanted hearts, the unregulated actomyosin ATPase of myosin is not different from myosin prepared from nonfailing hearts [38]–[40], further arguing that myosin itself is unlikely to be a primary target in the pathophysiology of cardiovascular disease.

These fiber mechanics measurements therefore allow us to speculate about the steps of muscle activation that limit the magnitude and rate of force production during ischemia. Since k_ATP_ is measured as the rate of force production from Ca^2+^-rigor, photolytic release of ATP results in an initial detachment of some rigor crossbridges, manifest as a brief and rapid decline in force followed by reattachment to activated thin filaments and force production (Fig. 4; [29], [30]). In the current study, k_ATP_ was measured to be significantly faster than k_Act_ (Fig. 3) but unaltered by ischemia. Taken together, the k_ATP_ and k_Act_ values suggest that the cycling rates of activated crossbridges were not rate-limiting, but rather that the transition of non-cycling crossbridges into activated, cycling crossbridges [28] was impaired. This is supported by the drop in Ca^2+^-rigor force in the ischemic fibres, demonstrating that the relationship between the relief of thin filament regulatory unit inhibition and initial crossbridge attachment [3] was impaired in ischemic fibers and limited the number of crossbridges that were able to transition from non-cycling to cycling states. In this regard, the data in this paper are best described by the activation and crossbridge cycling model described by Campbell [28]. This model stipulates that non-cycling crossbridges undergo a distinct activation transition to enter force bearing and non-force bearing cycling states. The separation of the activation transition from the cycling transitions is consistent with the k_ATP_ data, whereby ischemic fibers demonstrated reduced initial activation and attachment, manifest as a drop in Ca^2+^-rigor force, but unchanged k_ATP_ rate constants. Similar k_ATP_ in perfused and ischemic fibers suggests that those crossbridges that were able to enter the activated, cycling population demonstrated similar transition rates between force bearing and non-force bearing states. In contrast to k_ATP_, the rate constant of force production by Ca^2+^ also encompasses the rate of activation of non-cycling to cycling crossbridges. Therefore, the reduced k_Act_ in ischemic fibers suggests that the mechanism governing the rate of activation from non-cycling to cycling states, broadly represented as step 1 in Scheme 1, was impaired. The rate constant of force development after shortening, k_F_, closely resembled k_Act_ as expected, primarily because both techniques measure force production beginning from detached, largely non-cycling states that must progress through an activation transition prior to cycling between force bearing and non-force bearing states. This is consistent with prior demonstrations that a length release of activated fibers results in a substantially complete crossbridge detachment and increases myoplasmic Ca^2+^ [41], reflective of Ca^2+^ release from activated, Ca^2+^-bound thin filaments. This would be consistent with such regulatory units exiting the cycling population and/or returning to the blocked state [42], thereby needing to go through the activation transition prior to re-entering cycling states. Therefore, force redevelopment after the length release closely approximates force production following flash photolysis of NP-EGTA. The small numerical increase of k_F_ over k_Act_ likely reflects incomplete detachment or incomplete return of thin filaments to the blocked state due to a small number of persistent crossbridges that remain activated and in cycling states [43]. These two factors likely allow for a marginal number of crossbridges that remain activated to proceed rapidly to cycling states to effect a minor, incremental increase in the rate constant. Unlike k_F_, k_ATP_ was not altered by ischemia, suggesting that the reduced apparent rate constant of the Pi isomerization measured by length release experiments (Fig. 3) is due to this step being linked to the activation transition. Once the non-cycling to cycling activation transition is essentially complete (as in Ca^2+^-rigor), crossbridges readily cycle between force bearing and non-force bearing states with a higher apparent rate for k_1_ (Scheme 1), allowing k_ATP_ to be faster than both k_F_ and k_Act_. Therefore, at high Ca^2+^, thin filaments freely permit re-attachment of detached crossbridges, allowing them to readily transition between force bearing and non-force bearing states [28] at a rate that is likely limited by transitions subsequent to the Pi isomerization [44]. Therefore, in ischemic fibers, the decline in the rate constant of force activation following Ca^2+^ release is best described as a change in the rate of crossbridge activation into cycling states, which itself is a function of the molecular events that facilitate thin filament activation through an inter-related mechanism mediated by Ca^2+^ and strong crossbridge attachment [3], [28], [45], [46]. This scenario includes the possibility that the k_Act_ of ischemic fibers is slowed as a result of their reduced Ca^2+^ sensitivity, which may alter the cooperative recruitment of crossbridges into activated, cycling states.

To begin to decipher the underlying molecular basis for reduced contractility during ischemia, we concentrated on the regulatory troponin complex of the thin filament. Both TnT and TnI of the troponin complex are phosphorylated in *vivo* [17], leading us to examine possible changes in their phosphorylation states with ischemia. Analysis of TnT demonstrated a net dephosphorylation with ischemia that resulted in an increase in the abundance of the monophosphorylated form and an accompanying decrease in the diphosphorylated form (Fig. 5). At this time, it is difficult to assign a direct functional consequence to the reduction in TnT phosphorylation, as there are limited data documenting the *in vivo* sites of cardiac muscle TnT phosphorylation. A recent study suggested an *in vivo* site of phosphorylation within the first 29 amino terminal residues [47], and up to four sites may be phosphorylated *in vitro*, with Thr206 phosphorylation having a dominant, depressive effect on fiber contraction [48], [49]. As TnT forms intimate contacts with the tropomyosin filament, it is not unreasonable to assume that the change in phosphorylation status may influence the state of the thin filament and, in turn, the transition of crossbridges from non-cycling to attached, cycling states. Additional *in vitro* work using *in vivo* phosphorylated or dephosphorylated TnT will be required to address this mechanism.

To examine the possible impact of TnI phosphorylation, we focused on the adjacent residues Ser23 and 24, two sites that are especially relevant for β-adrenergic signaling. This signaling pathway may be altered in heart failure through a reduction in the content of PKA regulatory subunits, reduction in β-adrenergic receptor density, or desensitization of β-adrenergic receptors [50]–[52]. We examined the extent of Ser23/24 phosphorylation and noted a relative decrease in ischemic versus perfused fibers (Fig. 6A), consistent with previous observations [22], [50], [53], [54]. Although increased Ser23/24 phosphorylation of TnI is associated with decreased Ca^2+^ sensitivity under β-adrenergic stimulation [32], we observed decreased Ca^2+^ sensitivity with reduced Ser23/24 phosphorylation in this and a previous model [22], suggesting that TnI phosphorylation is not the sole contributor to the measured EC50 in ischemic fibers. This indicates that the integrated impact of altered TnI and TnT phosphorylation may uniquely alter the EC50 of muscle contraction. The decreased phosphorylation of TnI was accompanied by reduced MYBP-C phosphorylation (Fig. 7), supporting the notion that overall PKA activity was impaired in response to ischemia. Therefore, the endogenous PKA in perfused and ischemic fibers was activated by 8-Br-cAMP and the level of Ser23/24 phosphorylation of TnI was measured as an index of activity. As expected, 8-Br-cAMP increased Ser23/24 phosphorylation of TnI in fibers from perfused hearts (Fig. 6B), but there was no change in ischemic fibers (Fig. 6C). Interestingly, the protection provided by ischemic preconditioning has been suggested to involve early PKA activation during the preconditioning period [55], which may then counter this observed reduction of PKA activity during ischemia. The ability of 8-Br-cAMP to increase Ser23/24 phosphorylation in perfused fibers correlated with the increase in k_−1_ without a change in k_1_ (Fig. 8A), which we predict would be consistent with the lusitropic effect of β-adrenergic stimulation [56]. This effect of 8-Br-cAMP on the Pi isomerization rate was not observed in the ischemic fibers, consistent with Ser23/24 phosphorylation levels remaining unchanged, strongly suggesting that TnI phosphorylation is a modifier for this crossbridge transition. The 8-Br-cAMP treatment also resulted in similar relative increases in Fmax for perfused and ischemic fibers, although only the increases in ischemic fibers reached statistical significance (Fig. 8B). Nonetheless, the similar trends in force production following 8-Br-cAMP treatment of perfused and ischemic fibers contrast sharply with the observed changes in TnI phosphorylation. This divergence likely suggests that following 8-Br-cAMP treatment the factors responsible for controlling rate versus force are unique. This is further strengthened by observations from reperfused fibers demonstrating that force recovery was more readily achieved than rate recovery (Figs. 1, 2).

At this point, it is difficult to determine how the change in MYBP-C phosphorylation contributed to the accumulated results. Previous studies have reported reduced MYBP-C phosphorylation in models of altered myocardial blood flow, suggesting that this is a conserved target [8], [57], [58]. In a transgenic model, Stelzer *et al* [59] suggested that MYBP-C phosphorylation contributes to both systolic and diastolic function, and we cannot exclude such a contribution by MYBP-C phosphorylation in our mechanical experiments. Nonetheless, we would note that the change in k_−1_ and force following 8-Br-cAMP treatment of perfused fibers (Fig. 8) occurred without a significant change in total MYBP-C phosphorylation (Fig. 7). Although MYBP-C is a proven downstream target of β-adrenergic signaling, we do not think that the modest change in its total phosphorylation following 8-Br-cAMP treatment is anomalous. Previous studies have shown that following β-adrenergic stimulation with an agonist, incorporation of ^32^P into MYBP-C was significantly less in magnitude than other prominent targets such as TnI and phospholamban [32]. This is in contrast to methods whereby an excess of the PKA catalytic subunit is added to fibers [59], a strategy that is unlikely to preserve the physiological framework wherein changes in PKA subunit expression or phosphorylation state may dictate altered substrate selectivity [50], [60]. Our use of 8-Br-cAMP to activate the endogenous PKA better resembles agonist-dependent activation [32], suggesting that perfused fibers should demonstrate a larger change in TnI versus MYBP-C phosphorylation, as observed (Figs. 6,7). Further, given that MYBP-C is heavily phosphorylated at baseline [8], we are not surprised that the marginal increase in total MYBP-C phosphorylation was muted as compared to previous methods that employed the change of ^32^P incorporation over baseline [32].

In conclusion, the present study demonstrates that the actomyosin crossbridge cycle during ischemia is unique for a reduction in the forward rate constant of the isomerization of the Pi-bound actomyosin transition, which may be closely linked to the transition of non-cycling crossbridges to activated, cycling crossbridges. Additionally, the decrease in the rate of force activation with Ca^2+^ but not ATP is reflective of altered thin filament regulatory function and crossbridge recruitment during ischemia that modulates the apparent rates of activation of the actomyosin crossbridge cycle without impacting the intrinsic activity of the myosin enzyme. The *in vivo* model demonstrated reduced phosphorylation of TnT and TnI, suggesting that the integrated effect of altered troponin phosphorylation may be involved in the observed contractile response. Future studies will benefit from a focus on the thin filament and the post-translational state of regulatory proteins during ischemia in order to understand their individual and combined contributions to pathophysiological states marked by reduced contractility.
